# Freeze–Thaw Durability and Anisotropic Damage Evolution of 3D-Printed River-Sediment Engineered Cementitious Composites: Effects of Interlayer Interface Defects

**DOI:** 10.3390/ma19122559

**Published:** 2026-06-12

**Authors:** Lu Yin, Minjie Lv, Nan Ma, Fang Yuan, Jiajia Zhou, Chengfang Yuan

**Affiliations:** 1 School of Civil Engineering, Zhengzhou University, Zhengzhou 450001, China; yinlu@hnic.com.cn (L.Y.); lmj17839241782@gs.zzu.edu.cn (M.L.); zhouaf@zzu.edu.cn (J.Z.); 2 Henan Investment Group Co., Ltd., Zhengzhou 450003, China; manan@hnic.com.cn; 3 College of Civil and Transportation Engineering, Shenzhen University, Shenzhen 518060, China; fyuan@szu.edu.cn

**Keywords:** 3D-printed engineered cementitious composites, Yellow River sediment, freeze–thaw durability, interlayer interface defects, anisotropic damage evolution, relative dynamic elastic modulus, Weibull damage model

## Abstract

Freeze–thaw durability of 3D-printed engineered cementitious composites (3DP-ECC) is strongly affected by print-induced interlayer defects and anisotropy, particularly in cold regions. This study investigated Cast-ECC and Z-direction 3DP-ECC incorporating Yellow River sediment (YRS) as an equal-mass replacement for quartz sand at 0–100%. Compressive, three-point bending, and four-point bending tests, relative dynamic elastic modulus (RDME), XCT, MIP, SEM–EDS, and Weibull damage modeling were used to evaluate degradation up to 150 freshwater freeze–thaw cycles. Moderate YRS replacement (25–50%) improved particle packing, reduced visible defects, and refined the pore structure, thereby enhancing frost resistance. The R50 mixture showed the best residual performance: after 150 cycles, compressive strength decreased from 55 to 46 MPa in Cast-ECC and from 54 to 44 MPa in 3DP-ECC, corresponding to retention rates of 83.6% and 81.5%, respectively. The residual peak load in four-point bending of 3DP-ECC-R50 was 15.4% lower than that of Cast-ECC-R50, confirming the detrimental role of interlayer defects under loading perpendicular to the layers. RDME-based Weibull fitting described the overall damage evolution (R^2^ = 0.876–0.994), while XCT, MIP, and SEM–EDS indicated that interlayer discontinuities, pore-structure evolution, and local microstructural degradation governed anisotropic deterioration. The results support durability-oriented design of YRS-based 3DP-ECC in cold regions.

## 1. Introduction

Engineered Cementitious Composites (ECC), also referred to as Strain-Hardening Cementitious Composites (SHCC), have attracted considerable attention in the field of additive manufacturing in recent years owing to their superior tensile ductility and multiple cracking characteristics [1]. Several recent review papers [2,3,4,5] have pointed out that the combination of ECC and 3D printing technology has emerged as a rapidly advancing frontier research direction [6]. Experimental studies consistently demonstrate that 3D-printed ECC can exhibit tensile strain-hardening behavior and multiple microcrack propagation under loading [7,8,9,10]. With optimized fiber formulations, the tensile ductility (ultimate tensile strain) has been proven to exceed 5% [11]. Unlike traditional cementitious materials, ECC relies on the extensively investigated fiber bridging mechanism to achieve this pseudo-strain-hardening response [12,13]. The characteristic fine multiple cracking pattern produced by this mechanism fundamentally overcomes the inherent brittleness and catastrophic localized crack propagation commonly encountered in ordinary 3D-printed cementitious materials [14]. Consequently, ECC shows considerable promise for building-scale additive manufacturing [15].

However, when 3D-printed ECC is applied in cold regions, it will be subjected to long-term and repeated freeze–thaw cycles, making its durability a particularly critical issue. Compared with cast ECC, 3DP-ECC is fabricated through a layer-by-layer extrusion or deposition process, which inevitably introduces interlayer interfaces and an anisotropic microstructure [16]. These interfacial regions typically exhibit higher porosity and relatively weak bonding properties, and they can easily evolve into preferential paths for water penetration, migration, and stress concentration during freeze–thaw cycling, thereby accelerating microcrack initiation and propagation and causing a significant deterioration in the long-term service performance of the material. Studies on freeze–thaw damage mechanisms have demonstrated [17] that cementitious materials undergo a progressive degradation process under repeated freeze–thaw action. Therefore, revealing the damage evolution mechanisms of 3DP-ECC in freeze–thaw environments and exploring effective performance enhancement strategies are of considerable scientific significance and engineering value.

Utilizing solid waste to replace natural fine aggregates represents an important pathway for enhancing the sustainability of cementitious materials and optimizing the matrix microstructure. Yellow River sediment (YRS), as a silty aluminosilicate material with an enormous annual deposition volume, not only features a suitable particle size distribution but also possesses potential pozzolanic activity. Previous studies have shown [18] that an appropriate amount of YRS can improve matrix compactness and refine the pore structure through the “micro-filling effect” and optimization of particle gradation. Concerning the freeze–thaw durability of ECC, existing research has examined the durability evolution patterns of ECC [19] and the frost resistance of YRS-ECC [20,21]. However, most existing studies have focused on the influence of YRS on the mechanical properties of traditional cast ECC, while a systematic understanding of its application in 3DP-ECC—particularly regarding the regulatory mechanisms governing the interlayer interface structure and freeze–thaw damage behavior—remains lacking [18]. Notably, while YRS incorporation modifies the pore structure of the matrix, it is highly likely to produce coupling effects with the anisotropy induced by the 3D printing process; the influence of these effects on freeze–thaw durability still requires in-depth investigation.

Furthermore, concerning freeze–thaw damage characterization, most existing work has adopted empirical indicators or homogeneous material models, which fail to adequately account for the inherent anisotropic characteristics of 3DP-ECC and their influence on the damage evolution path. Therefore, it is urgent to establish a statistical evolution model that can reflect the anisotropic damage law of 3DP-ECC, so as to enable accurate life prediction of the material under freeze–thaw environments. Previous researchers have applied the Weibull distribution to the reliability analysis of freeze–thaw damage in fiber-reinforced concrete [22], providing a methodological basis for the model construction in this study.

Although recent studies have improved the understanding of 3DP-ECC in terms of printability, fiber orientation, strain-hardening behavior, and mechanical anisotropy, most existing research has focused on fresh-state performance or short-term mechanical response. Quantitative durability evaluation under freeze–thaw exposure remains limited. In particular, the role of interlayer interfaces as preferential channels for water ingress, stress concentration, and crack propagation has not been sufficiently linked to stiffness degradation and residual strength loss.

Existing studies on ECC freeze–thaw durability have mainly examined conventional cast specimens and evaluated degradation using mass loss, residual mechanical properties, and relative dynamic elastic modulus (RDME). However, these studies generally treat ECC as a homogeneous or quasi-isotropic composite. This assumption is not applicable to 3DP-ECC, in which layer-by-layer extrusion inevitably introduces interlayer voids, weak bonding regions, and direction-dependent fiber distribution. Therefore, the freeze–thaw damage mechanisms identified in cast ECC cannot be directly extended to printed ECC without considering printing-induced interlayer anisotropy.

Studies on YRS and other fine sediment materials have shown that moderate replacement can improve matrix compactness by optimizing particle packing and refining the pore structure. Nevertheless, excessive replacement may increase matrix heterogeneity, reduce cohesion, and accelerate durability loss. These inconsistent observations indicate that the effect of YRS depends not only on its micro-filling effect, but also on its interaction with pore connectivity, fiber dispersion, and interfacial bonding. In 3DP-ECC, this interaction is further complicated by printing-induced interlayer defects and anisotropic fiber orientation.

In addition, although Weibull-based models have been used to describe freeze–thaw damage in cementitious materials, most existing models are not explicitly connected with anisotropic interlayer damage or microstructural evidence. Few studies have integrated RDME degradation, residual strength loss, pore-structure evolution, SEM–EDS characterization, and probabilistic damage modeling to explain the freeze–thaw deterioration of 3DP-ECC. In particular, direct comparisons between Cast-ECC and Z-direction 3DP-ECC with identical mix proportions remain scarce. This lack of coupled quantitative evidence limits the understanding of how printing-induced anisotropy governs freeze–thaw damage evolution.

Therefore, the originality of this study lies in establishing a coupled framework that links YRS-based material design, interlayer anisotropy, microstructural degradation, and Weibull damage evolution. By comparing Cast-ECC and Z-direction 3DP-ECC with identical YRS replacement ratios under up to 150 freeze–thaw cycles, this study clarifies how interlayer defects influence moisture ingress, crack propagation, stiffness degradation, and residual mechanical performance. The findings provide a mechanistic basis for the durability-oriented design of 3D-printed ECC structures in cold regions.

Based on the above critical analysis, this study aims to clarify the coupled effects of YRS replacement and printing-induced interlayer anisotropy on the freeze–thaw durability of ECC. Unlike previous studies that separately examined mechanical properties, durability indicators, or microstructural characteristics, this work integrates macroscopic performance degradation, RDME-based damage quantification, XCT, MIP, SEM–EDS microstructural evidence, and Weibull statistical modeling. The main original contributions are as follows:

(1) Quantitatively comparing the freeze–thaw durability of Cast-ECC and Z-direction 3DP-ECC with identical YRS replacement ratios, thereby isolating the effect of printing-induced interlayer anisotropy.

(2) Revealing how interlayer interface defects govern moisture ingress, crack propagation, stiffness degradation, and strength loss in 3DP-ECC under repeated freeze–thaw cycles.

(3) Clarifying the dual role of YRS in improving matrix compactness at moderate replacement levels and aggravating microstructural heterogeneity at excessive replacement levels.

(4) Establishing a Weibull-based freeze–thaw damage model using RDME degradation and linking the model parameters with microstructural mechanisms supported by XCT, MIP, and SEM–EDS observations.

(5) Developing a coupled mechanism that connects material design, interlayer anisotropy, microstructural deterioration, and statistical damage evolution for the durability-oriented design of 3D-printed ECC in cold regions.

## 2. Materials and Methods

### 2.1. Materials and Mix Proportion

The primary binder used in this study was Ordinary Portland Cement (P.O. 42.5), supplied by Zhengzhou Tianrui Cement Co., Ltd., Zhengzhou, Henan Province, China, whose technical specifications are summarized in Table 1.

The supplementary cementitious material used was Grade II fly ash obtained from Hannuo Filter Material Co., Ltd., Gongyi, Henan Province, China. The chemical compositions of the two cementitious materials (cement and fly ash) are presented in Table 2. The spherical particle morphology of the fly ash is conducive to improving the fluidity of the fresh paste, reducing the matrix fracture toughness, and thereby exerts a positive effect on the ductility enhancement of ECC. The main technical indicators of Yellow River sediment (YRS) are summarized in Table 3.

The fine aggregate used was Yellow River sediment (YRS) sourced from Puyang, Henan Province, China, which is a silty material. Particles finer than 75 μm accounted for 81.4% of the total mass; its apparent density was 2646 kg/m^3^, bulk density was 1419 kg/m^3^, saturated surface-dry water absorption was 2.1%, and specific surface area was 0.434 m^2^/g. Meanwhile, 80–120 mesh quartz sand (Henan Zhongbang Environmental Technology Co., Ltd., Zhengzhou, Henan Province, China) was incorporated to optimize the performance. Hydroxypropyl methylcellulose (HPMC-20, Shanghai Chenqi Chemical Technology Co., Ltd., Shanghai, China) was adopted as a thickening agent. A CQJ-JSS type polycarboxylate-based high-performance superplasticizer (Shanghai Chenqi Chemical Technology Co., Ltd., Shanghai, China) was used to improve the workability of the fresh mixture. Polyethylene (PE) fiber, supplied by Henan Jingwei Building Materials Co., Ltd., Henan Province, China, was used as the reinforcing material, and its main technical parameters are listed in Table 4. Attapulgite (ATP, 1250 mesh), supplied by Lingshou County Shanchuan Mineral Products Processing Plant, Hebei Province, China, was used in this study, and its characteristics are shown in Table 5.All 3DP-ECC specimens were fabricated using a gantry-based extrusion 3D printing system equipped with a circular nozzle with a diameter of 20 mm. The mix design for 3D YRS-ECC printing is shown in Table 6.

### 2.2. Specimen Design and Fabrication

All 3DP-ECC specimens were fabricated using an extrusion-based 3D printing system equipped with a circular nozzle with a diameter of 20 mm. Beam specimens for flexural tests were designed with dimensions of 40 mm × 40 mm × 160 mm, while cubic specimens for compressive tests had dimensions of 50 mm × 50 mm × 50 mm. The printed specimens were extracted from Z-direction printed build plates, in which the loading direction was perpendicular to the printed layers. The nominal layer thickness was approximately 10 mm.

During fabrication, the fresh ECC mixture was extruded layer by layer to form a compact printed build plate. After printing, the cutting positions were marked, and standard beam and cube specimens were obtained using a high-pressure water-jet cutting machine using a laboratory high-pressure water-jet cutting system. This procedure was adopted to ensure dimensional accuracy, specimen consistency, and comparability among different mixture groups. The experimental grouping scheme is summarized in Table 7. In this table, the replacement ratio refers to the mass percentage of Yellow River sediment (YRS) used to replace quartz sand in the ECC mixture. Specifically, R0, R25, R50, R75, and R100 represent YRS replacement levels of 0%, 25%, 50%, 75%, and 100%, respectively. The paired groups refer to Cast-ECC and Z-direction 3DP-ECC specimens prepared with the same YRS replacement ratio, allowing direct comparison between cast and printed specimens.

This study focused on the freeze–thaw durability and anisotropic damage evolution of hardened 3DP-ECC rather than on the systematic optimization of fresh-state printability. Before specimen preparation, the mixtures were preliminarily adjusted to ensure continuous extrusion, stable filament formation, and successful layer deposition under the selected printing conditions. To maintain comparability among different YRS replacement ratios, the same printing procedure and specimen preparation method were used for all 3DP-ECC mixtures. Therefore, buildability, extrudability, open time, rheological parameters, and thixotropic recovery were not independently quantified in the present study.

For each test condition, three replicate specimens were prepared and tested to ensure repeatability. The number of test conditions in Table 7 was determined by five YRS replacement ratios, two specimen types, and four freeze–thaw exposure levels, resulting in a total of 40 test conditions for each performance index. Accordingly, 120 specimens were tested for each mechanical performance index, and the reported results are presented as the average values of three replicate specimens.

### 2.3. Mechanical Properties Test

**Figure 1:**
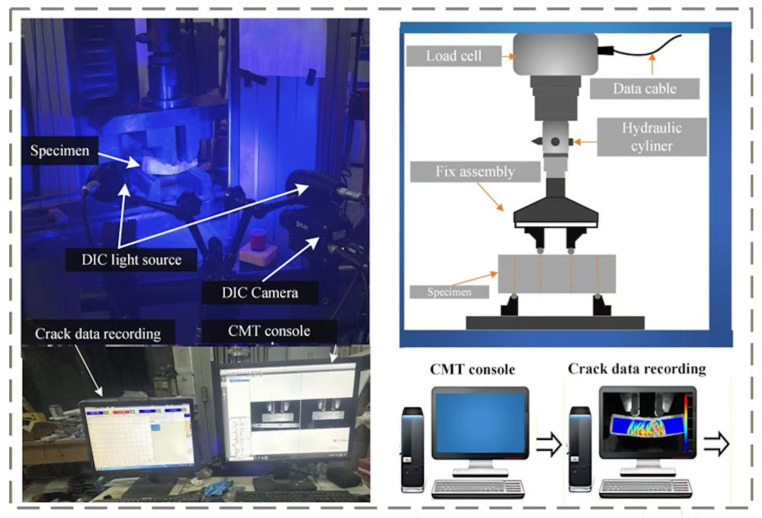
Testing method for four-point bending test.

**Figure 2:**
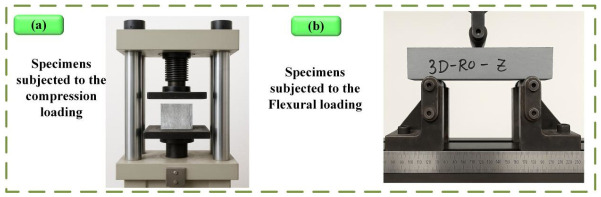
Testing method for compressive and flexural strength: (**a**) compressive loading test setup; (**b**) flexural loading test setup.

The mechanical properties of the cast and 3D-printed ECC specimens were evaluated through four-point bending, compressive, and three-point bending tests, respectively. The four-point bending tests were performed on a 200 kN servo-hydraulic universal testing machine at a displacement rate of 0.5 mm/min, with a clear span of 120 mm; the symmetric two-point loading produced a pure bending zone to eliminate shear effects. During the test, a digital image correlation (DIC) system was employed to monitor crack evolution and full-field strain. Compressive strength tests were conducted in accordance with GB/T 50081 2019 [23] and ASTM C109 [24], with three parallel specimens tested in each group and the average value adopted. Three-point bending tests were carried out following GB/T 17671-2021 [25]. Flexural strength and cracking behavior were evaluated by recording the load–deflection curves, enabling a direct comparison of the flexural performance between Cast-ECC and 3DP-ECC. The test setups are illustrated in Figure 1 and Figure 2a,b. For all mechanical tests, three replicate specimens were tested for each mixture, specimen type, and freeze–thaw exposure condition. The average value of the three replicate specimens was used for subsequent analysis. To ensure the reliability of the experimental data, specimens with obvious casting or printing defects, severe dimensional deviation, damage during demolding, cutting, curing, or freeze–thaw conditioning, and abnormal failure modes unrelated to the designed loading condition were excluded from the analysis. If an individual result showed a clear deviation from the other replicate results, the specimen was rechecked, and the result was excluded only when an identifiable preparation or testing defect was confirmed.

### 2.4. Freeze–Thaw Resistance Test

Freeze–thaw durability tests were conducted on both Cast-ECC and 3DP-ECC specimens in accordance with GB/T 50082-2009 [26]. Before freeze–thaw exposure, all specimens were cured to the designed age and then immersed in freshwater to achieve a saturated condition. The tests were performed in a programmable freeze–thaw chamber using freshwater as the exposure medium. During cycling, the chamber operated automatically within a temperature range of −20 °C to 7 °C.

The freeze–thaw cycles were interrupted at 0, 25, 50, 75, 100, and 150 cycles for mass measurement and dynamic elastic modulus measurement. Mechanical tests were conducted at 0, 50, 100, and 150 cycles to evaluate residual compressive and flexural performance. Before each measurement, the specimens were removed from the chamber, and the surface water was gently wiped off to minimize the influence of free surface moisture. The same freeze–thaw procedure was applied to both Cast-ECC and 3DP-ECC specimens to ensure comparability between the two fabrication methods. The designations 0 N, 50 N, 100 N, and 150 N denote the corresponding numbers of freeze–thaw cycles, as summarized in Table 8.

It should be noted that the programmable freeze–thaw chamber operated according to a preset automatic program. However, the chamber temperature log and the resonance-frequency records were not archived during testing. Therefore, the exact cooling rate, heating rate, duration of each freezing and thawing stage, and excitation frequency used for dynamic modulus measurement cannot be retrospectively reported. Accordingly, the present results are interpreted as cycle-number-based comparative durability data rather than as a complete thermal-history-based freeze–thaw analysis.

**Figure 3:**
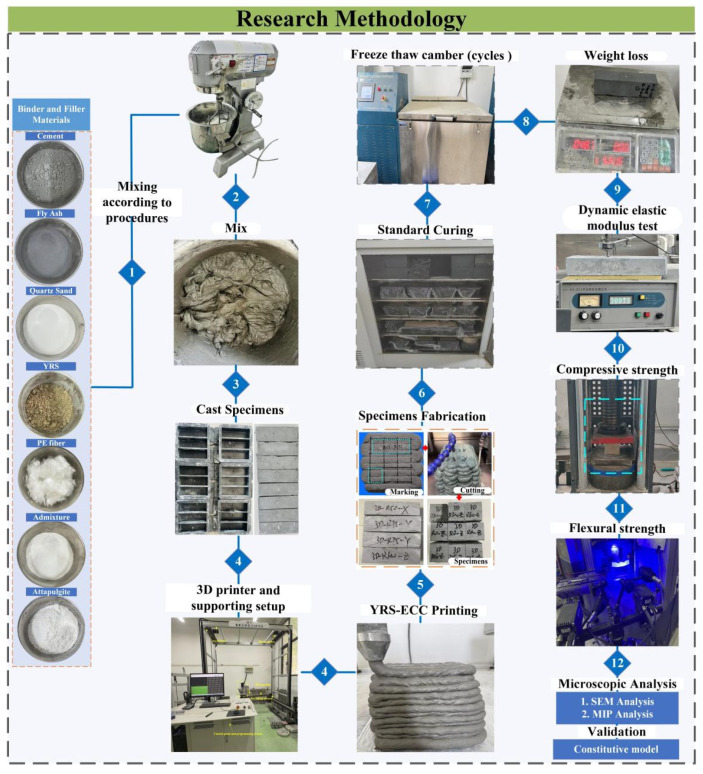
The complete research methodology.

**Figure 4:**
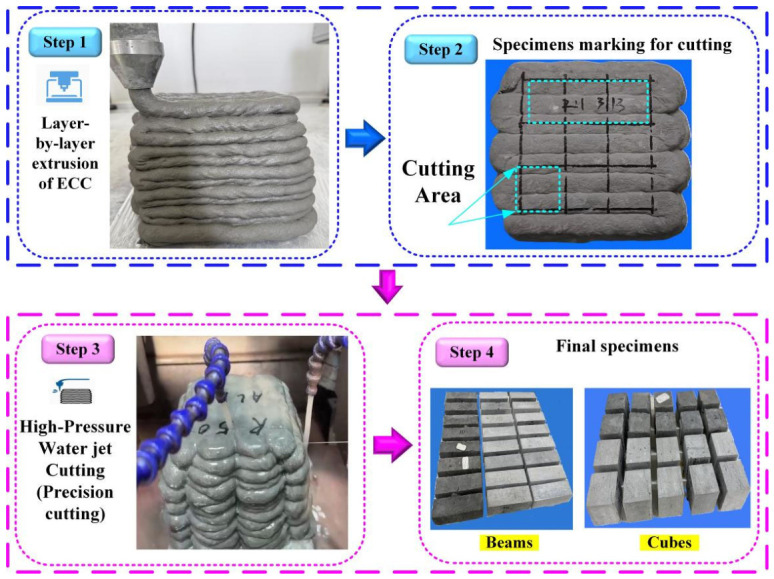
Fabrication of 3DP-ECC specimens.

**Figure 5:**
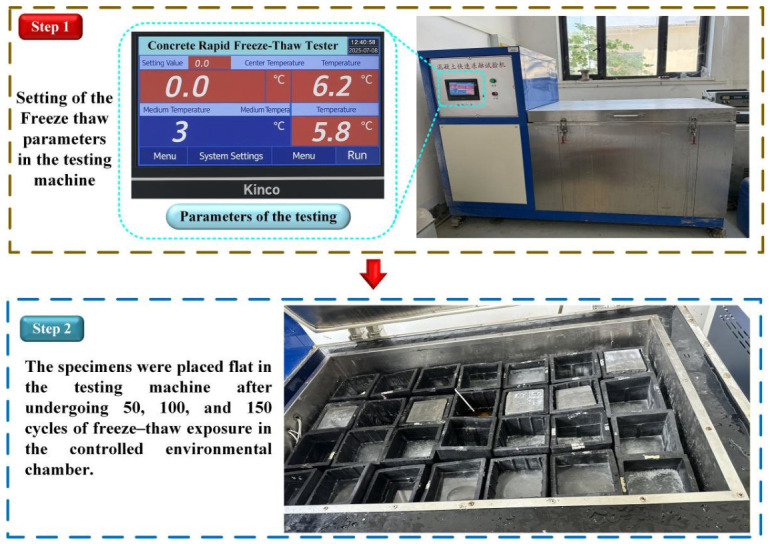
The complete experimental setup for the freeze–thaw testing.

After the specified numbers of freeze–thaw cycles, the mass loss rate, relative dynamic modulus of elasticity, and residual mechanical properties were measured to evaluate the degradation of ECC under repeated freeze–thaw exposure. Figure 3 presents the overall research methodology adopted in this study. The fabrication and specimen preparation procedures of the 3DP-ECC specimens are illustrated in Figure 4, while Figure 5 shows the freeze–thaw testing setup and the dynamic modulus measurement procedure.

The relative dynamic modulus of elasticity (RDME) was calculated as the ratio of the dynamic modulus after a given number of freeze–thaw cycles to the initial dynamic modulus before freeze–thaw exposure, as expressed in Equation (1):

(1) $$M_{rd}=\frac{M_{n}}{M_{0}}$$

Here, the relative dynamic modulus of elasticity ($M_{rd}$) is defined as the dynamic modulus after n freeze–thaw cycles ($M_{n}$) divided by the initial modulus ($M_{0}$), both expressed in GPa.

In this study, freeze–thaw durability was evaluated using mass loss rate, RDME, and residual mechanical properties. These indicators were selected because they directly reflect surface deterioration, internal stiffness degradation, and load-bearing capacity loss under repeated freeze–thaw action. Other transport-related durability indicators, including water absorption, sorptivity, permeability, scaling resistance, chloride resistance, and salt freeze–thaw resistance, were not included in the present experimental program. Therefore, the durability discussion in this study is limited to freshwater freeze–thaw degradation and the associated mechanical and microstructural damage evolution.

For each freeze–thaw condition, three replicate specimens were prepared for each YRS replacement ratio and specimen type. The mass loss rate, relative dynamic modulus of elasticity, and residual mechanical properties were determined from the average values of the three replicate specimens. This experimental design was adopted to minimize the influence of specimen-to-specimen variability and to ensure the repeatability of the freeze–thaw durability evaluation.

The present study focused on freshwater freeze–thaw degradation and the associated mechanical and microstructural damage evolution of Cast-ECC and 3DP-ECC. Transport-related durability indicators, including water absorption, sorptivity, permeability, salt scaling resistance, chloride resistance, and salt freeze–thaw resistance, were beyond the scope of the present experimental program. Therefore, the conclusions of this study should be limited to freshwater freeze–thaw durability and should not be generalized to all durability environments.

### 2.5. Microstructural Characterization

X-ray computed tomography (XCT) was used to examine the internal void morphology and particle distribution of selected Cast-ECC and 3DP-ECC specimens. Three-dimensional reconstruction and image segmentation were performed using VGSTUDIO MAX 2025.4 software (Volume Graphics GmbH, Heidelberg, Germany).Based on grayscale contrast and morphological characteristics, internal voids, fine aggregates, and hydration products were identified to evaluate the influence of YRS replacement and the printing process on internal defects and anisotropic microstructure.

Mercury intrusion porosimetry (MIP) was conducted to quantify the pore-structure characteristics of representative specimens, including pore-size distribution, total porosity, average pore diameter, median pore diameter, and the relative proportions of pores within different size ranges. The MIP results were used to support the interpretation of matrix densification, pore refinement, and freeze–thaw-induced stiffness degradation.

SEM–EDS analysis was performed to provide local microstructural evidence of interfacial morphology, hydration-product distribution, microcrack development, and elemental redistribution before and after freeze–thaw exposure. It should be noted that SEM–EDS observations were conducted on selected regions and therefore should be interpreted as local evidence supporting the macroscopic degradation trends, rather than as a fully quantitative characterization of the entire matrix or pore system.

The combined XCT, MIP, and SEM–EDS analyses were used to link visible internal defects, pore-structure parameters, interlayer morphology, and freeze–thaw damage evolution. These complementary techniques enabled the freeze–thaw degradation of Cast-ECC and 3DP-ECC to be interpreted from both macro- and microstructural perspectives. However, XRD, FTIR, and TGA/DTG analyses were not included in the present experimental program. Therefore, the chemical phase evolution and quantitative decomposition of hydration products were not characterized in this study and should be investigated in future work.

## 3. Results and Discussion

The following section discusses the freeze–thaw degradation behavior of Cast-ECC and 3DP-ECC from four coupled perspectives: degradation rate, mechanical retention, microstructural deterioration, and statistical damage evolution. In addition to describing the trends shown in the figures, quantitative indices including strength loss rate, RDME degradation rate, flexural stiffness reduction, Weibull model parameters, ANOVA significance, and C.V.-based repeatability are used to interpret the mechanisms governing freeze–thaw damage.

### 3.1. Mass Loss and Dynamic Modulus Evolution Under Freeze–Thaw Cycles

The coupled evolution of mass loss and relative dynamic modulus of elasticity (RDME) indicates that freeze–thaw deterioration was governed by two sequential but interrelated mechanisms: surface material scaling and internal stiffness degradation. During the early stage of freeze–thaw exposure, mass loss remained limited, suggesting that external erosion was not the dominant damage mode. In contrast, the gradual decrease in RDME indicates that internal microcrack initiation and stiffness degradation occurred before visible surface deterioration. Therefore, RDME can be regarded as a more sensitive indicator than mass loss for detecting early-stage freeze–thaw damage.

**Figure 6:**
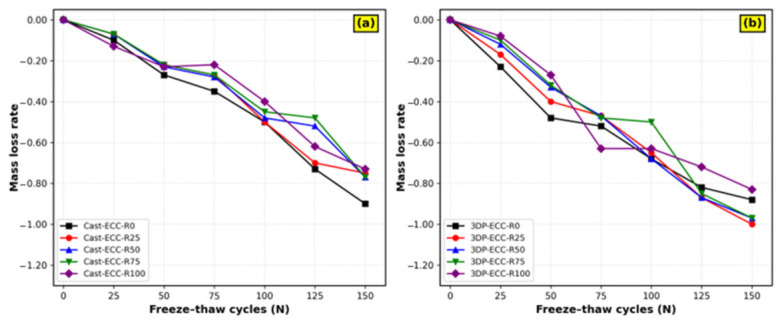
Changes in mass for 3DP-ECC after repeated freeze–thaw cycles. (**a**) Cast; (**b**) 3DP.

As shown in Figure 6, during the first 50 freeze–thaw cycles, the mass loss of all mixtures remained below 0.30%, indicating that severe surface scaling had not yet occurred. However, this limited mass loss does not imply the absence of internal deterioration, because RDME had already begun to decrease during the same period. This difference suggests that the initial freeze–thaw damage was mainly controlled by pore-water expansion, internal microcrack initiation, and early degradation of the matrix skeleton, rather than by external material loss. The relatively slow deterioration at this stage may be partly attributed to the continued hydration under saturated conditions and the fine-particle filling effect of YRS, which can improve local compactness and partially offset early freeze–thaw damage.

**Figure 7:**
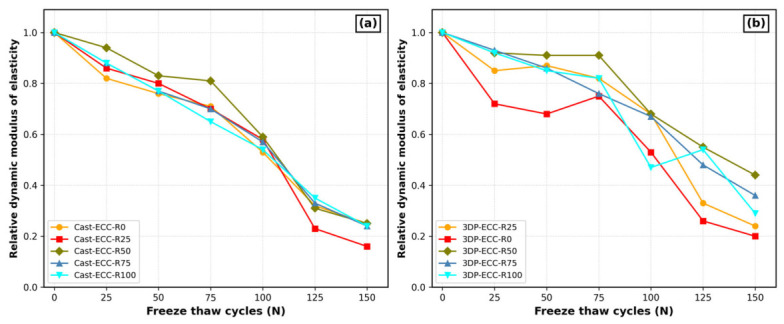
Relative dynamic modulus of elasticity after repeated freeze–thaw cycles. (**a**) Cast; (**b**) 3DP.

Figure 7 shows the RDME evolution of Cast-ECC and 3DP-ECC specimens with different YRS replacement ratios under freshwater freeze–thaw conditions. Changes in RDME reflect the initiation, propagation, and coalescence of internal microcracks induced by cyclic freezing and thawing. Compared with mass loss, RDME provides a more direct indication of internal stiffness degradation and damage accumulation.

To avoid a purely descriptive interpretation of the mass loss and RDME curves, the RDME-based degradation rate was further quantified as follows:

(2) $$V_{D}=\frac{{1-M}_{rd,150}}{150}$$

where $V_{D}$ is the average RDME-based degradation rate per freeze–thaw cycle, and $M_{rd,150}$ is the RDME after 150 freeze–thaw cycles. After 150 cycles, the RDME values of Cast-ECC specimens ranged from 0.16 to 0.25, corresponding to an average degradation rate of approximately 0.50–0.56% per cycle. For 3DP-ECC specimens, the residual RDME values ranged from 0.20 to 0.44, corresponding to an average degradation rate of approximately 0.37–0.53% per cycle. These results indicate that freeze–thaw deterioration was not governed solely by the number of cycles, but also by the combined effects of matrix compactness, pore connectivity, YRS replacement ratio, and printing-induced interlayer defects.

The lower degradation rates observed in the R25 and R50 mixtures can be attributed to the improved particle packing and reduced capillary connectivity induced by moderate YRS incorporation. Moderate YRS replacement refined the pore structure and reduced the continuity of water migration channels, thereby delaying the development of internal freeze–thaw damage. In contrast, the higher degradation observed in the R0 and R100 mixtures indicates that both insufficient fine-particle filling and excessive fine-particle content can increase microstructural vulnerability. For the R0 mixture, the lack of optimized particle gradation resulted in a relatively loose matrix structure. For the R100 mixture, excessive fine particles increased matrix heterogeneity and pore connectivity, thereby accelerating freeze–thaw damage [27]. Therefore, the influence of YRS on freeze–thaw resistance was nonlinear rather than monotonic.

By 100 freeze–thaw cycles, both mass loss and RDME reduction became more pronounced than those observed after 50 cycles, reflecting the progressive propagation and interconnection of internal microcracks as well as the deterioration of the pore structure [17,28]. Among all mixtures, the R25 and R50 groups exhibited relatively lower mass loss and higher RDME retention, confirming that an appropriate amount of YRS can improve freeze–thaw resistance by optimizing particle gradation, refining the pore structure, and reducing capillary pore connectivity.

Compared with Cast-ECC, the freeze–thaw degradation of 3DP-ECC was more strongly affected by interlayer defects and anisotropic crack paths. The layer-by-layer deposition process introduced weak interfaces and local pore discontinuities, which served as preferential channels for water migration and stress concentration under freeze–thaw cycles. Therefore, even when the overall RDME retention of some 3DP-ECC mixtures appeared comparable to that of Cast-ECC, their damage mechanisms were fundamentally different. In 3DP-ECC, stiffness loss was more likely to initiate and propagate along the interlayer regions, whereas in Cast-ECC, damage was more uniformly distributed within the matrix. This confirms that weak interlayer interfaces and printing-induced pore discontinuities accelerate freeze–thaw damage evolution in 3DP-ECC [18,19,28].

### 3.2. Evolution Law of Compressive Strength

**Figure 8:**
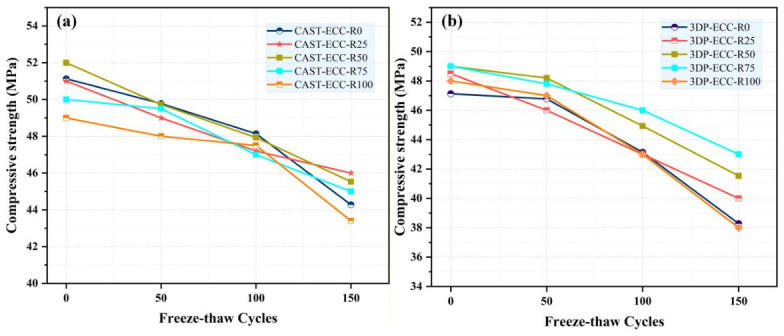
Compressive strength variation: (**a**) Cast-ECC; (**b**) 3DP-ECC (Z Direction).

The evolution of compressive strength indicates that freeze–thaw damage was governed not only by the number of freeze–thaw cycles, but also by aggregate gradation and specimen fabrication method. The compressive strength variation of Cast-ECC and 3DP-ECC specimens with different YRS replacement ratios under freeze–thaw cycles is shown in Figure 8. The reduction in compressive strength after freeze–thaw exposure can be attributed to the combined effects of matrix microcracking, weakening of fiber–matrix bonding, and deterioration of load-transfer continuity.

In the initial state, Cast-ECC exhibited compressive strengths of 49–52 MPa, whereas 3DP-ECC showed slightly lower values of 47.13–49 MPa. This difference suggests that the layer-by-layer printing process introduced structural discontinuities and weak interlayer regions even before freeze–thaw exposure. Among all mixtures, the R50 mixture achieved the highest compressive strength for both Cast-ECC and 3DP-ECC, indicating that 50% YRS replacement provided the most favorable balance among micro-filling, particle packing, and matrix cohesion. Appropriate YRS incorporation can optimize particle packing, refine the pore structure, and improve interfacial bonding, thereby enhancing matrix compactness and mechanical performance [21,27].

**Figure 9:**
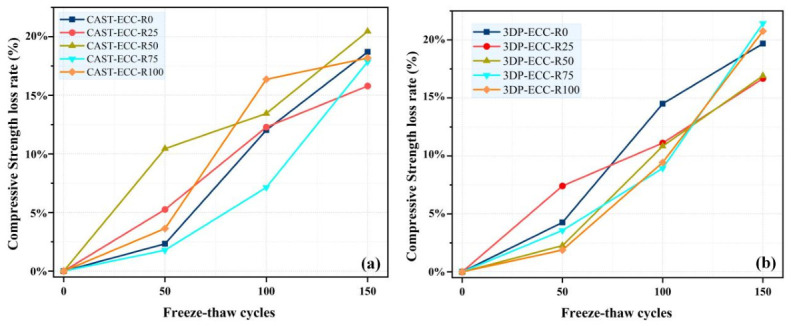
Compressive strength rate loss: (**a**) Cast-ECC; (**b**) 3DP-ECC (Z Direction).

During the early freeze–thaw stage of 0–50 cycles, the reduction in compressive strength remained relatively moderate. This limited degradation indicates that the internal structure of the specimens was still comparatively stable at this stage. Continued hydration under saturated conditions and the fine-particle filling effect of YRS may have partially compensated for the damage caused by freezing-induced pore-water expansion. The compressive strength loss of all specimens is presented in Figure 9.

As the number of freeze–thaw cycles increased from 50 to 100, strength deterioration became more pronounced. After 150 freeze–thaw cycles, the degradation of the internal structure became more severe. The volume expansion of freezing water generated hydraulic pressure within the pore system, promoting microcrack initiation, propagation, and coalescence in the matrix [17,28]. This progressive internal damage weakened the matrix skeleton and reduced the load-bearing capacity of the specimens.

Compared with 3DP-ECC, Cast-ECC exhibited better frost resistance because of its relatively homogeneous and monolithic structure. In contrast, the inherent interlayer interfaces and extrusion-induced pores in 3DP-ECC increased the continuity of water migration paths and promoted localized stress concentration under freeze–thaw action, thereby accelerating strength degradation [18,19].

After 150 freeze–thaw cycles, the residual compressive strength followed the order R50 > R25 > R0 > R75 > R100. This ranking demonstrates the nonlinear effect of YRS replacement on compressive performance. Moderate YRS replacement improved particle packing and reduced pore connectivity, thereby delaying frost-induced crack coalescence. In contrast, excessive YRS replacement increased the fine-particle content, water demand, and matrix heterogeneity, which weakened matrix continuity and accelerated freeze–thaw-induced strength loss. A YRS replacement ratio of 25–50% was therefore more effective in optimizing particle gradation and regulating the pore structure [18,20]. Excessively high YRS content may impair matrix cohesion and increase microvoid formation, leading to aggravated performance degradation [20].

In addition, PE fibers contributed to the stabilization of compressive performance by bridging microcracks and restraining crack propagation during freeze–thaw exposure. The statistical reliability of the compressive strength results was further supported by the ANOVA results presented in Section 3.7. The Cast-ECC and 3DP-ECC compressive strength models showed *p*-values of 0.0002 and 0.0014, respectively, indicating that aggregate composition had a statistically significant effect on compressive performance.

### 3.3. Bending Properties and Crack Evolution Behavior

The flexural response was more sensitive to freeze–thaw damage than compressive strength because bending failure is primarily governed by tensile crack initiation, fiber bridging, and interfacial load transfer. Before freeze–thaw exposure, all specimens exhibited typical ECC multiple-cracking and fiber-bridging behavior, indicating that the PE fibers effectively redistributed tensile stress and delayed localized failure [12,13]. However, repeated freeze–thaw cycles progressively widened microcracks and intensified strain localization, reducing the ability of fibers to transfer stress across cracks. As a result, both flexural stiffness and peak load gradually decreased with increasing freeze–thaw cycles.

Before freeze–thaw cycling, the R50 mixture exhibited the best initial flexural performance among all mixtures. The peak load and corresponding deflection of Cast-ECC-R50 reached 9.89 kN and 7.5 mm, respectively, while those of 3DP-ECC-R50 were 8.5 kN and 6.5 mm. This superior performance can be attributed to the optimized particle packing, improved matrix compactness, and enhanced fiber–matrix interaction induced by moderate YRS incorporation. In contrast, excessive YRS replacement in the R75 and R100 mixtures increased matrix heterogeneity and reduced cohesion, resulting in lower flexural capacity and earlier post-peak softening.

With increasing freeze–thaw cycles, all specimens exhibited gradual reductions in flexural capacity, stiffness, and ductility. The failure pattern also shifted from dispersed multiple cracking to more localized crack propagation. This transition was mainly caused by freezing-induced pore-water expansion, which promoted microcrack initiation and propagation and weakened the fiber–matrix interface. Compared with Cast-ECC, 3DP-ECC showed more severe strain localization, with cracks preferentially developing along or near the interlayer interfaces [18,20]. This behavior indicates that the printing-induced anisotropic microstructure accelerated frost-induced flexural deterioration.

PE fibers continued to play an important crack-bridging role during freeze–thaw exposure by limiting crack opening and delaying localized failure [12,13]. However, their bridging efficiency gradually decreased as freeze–thaw damage accumulated. Mixtures containing 25–50% YRS maintained more stable flexural performance because of their refined pore structure and improved matrix compactness. In contrast, the R75 and R100 mixtures experienced more severe degradation due to higher matrix heterogeneity, increased water migration paths, and faster microcrack development.

**Figure 10:**
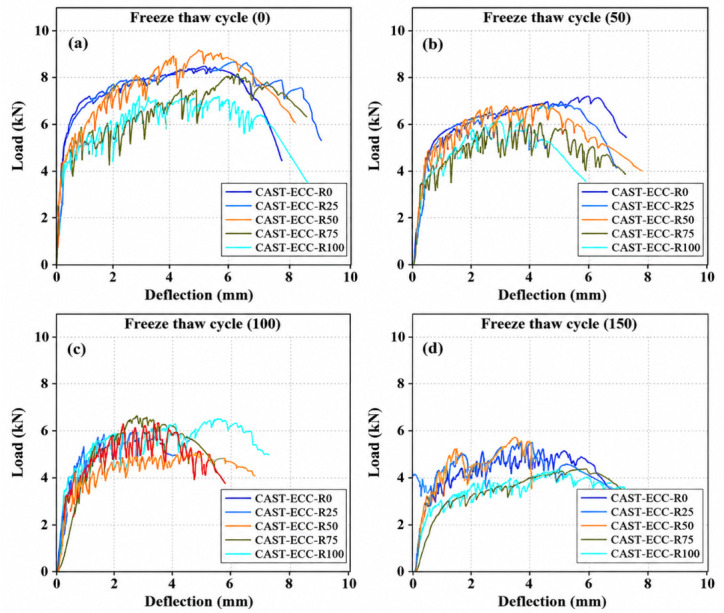
Durability results of the four-point bending performance of the Cast-ECC: (**a**) 0 freeze–thaw cycles; (**b**) 50 freeze–thaw cycles; (**c**) 100 freeze–thaw cycles; and (**d**) 150 freeze–thaw cycles.

**Figure 11:**
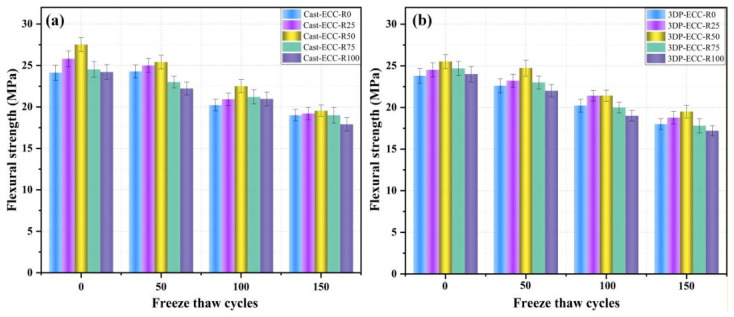
Flexural strength of specimens with varying YRS contents under successive freeze–thaw cycles: (**a**) Cast-ECC; (**b**) 3DP-ECC (Z direction).

**Figure 12:**
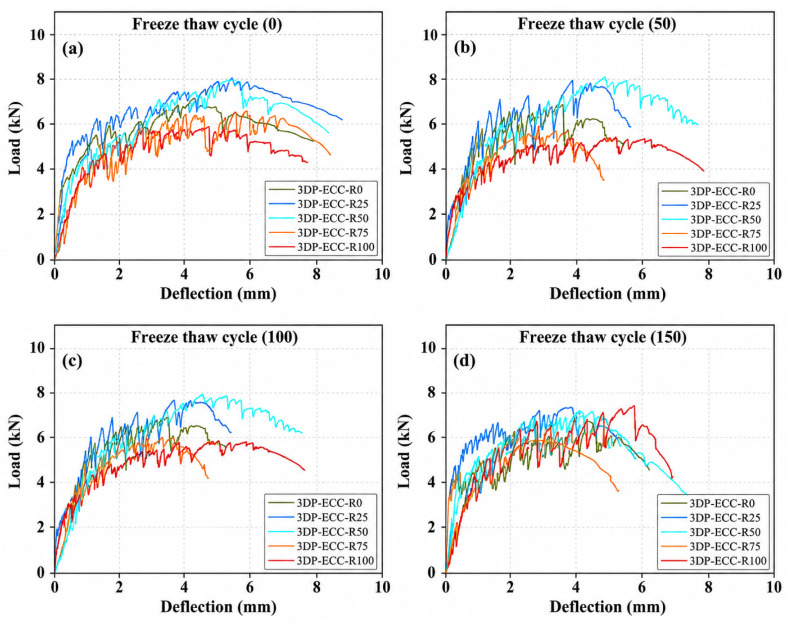
Load–deflection curves of 3DP-ECC specimens under four-point bending after different freeze–thaw cycles: (**a**) 0 freeze–thaw cycles; (**b**) 50 freeze–thaw cycles; (**c**) 100 freeze–thaw cycles; and (**d**) 150 freeze–thaw cycles.

**Figure 13:**
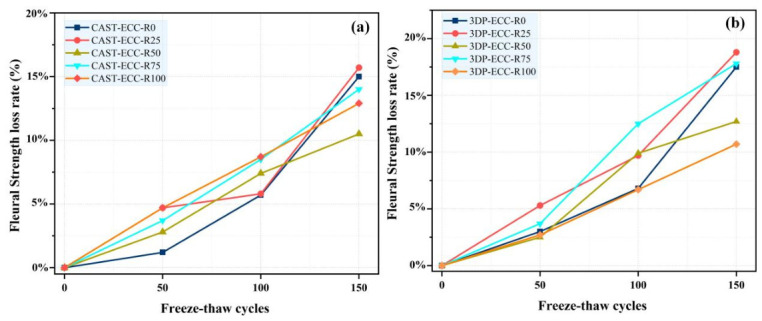
Flexural-strength loss rate of specimens with varying YRS contents under successive freeze–thaw cycles: (**a**) Cast-ECC; (**b**) 3DP-ECC (Z direction).

The four-point bending performance of Cast-ECC specimens under different freeze–thaw cycles is shown in Figure 10. As further summarized in Figure 11, the flexural strength of both Cast-ECC and 3DP-ECC varied with YRS replacement ratio and freeze–thaw exposure. Figure 12 presents the four-point bending performance of 3DP-ECC specimens, while Figure 13 shows the corresponding flexural-strength loss rates of Cast-ECC and 3DP-ECC specimens under successive freeze–thaw cycles.

**Figure 14:**
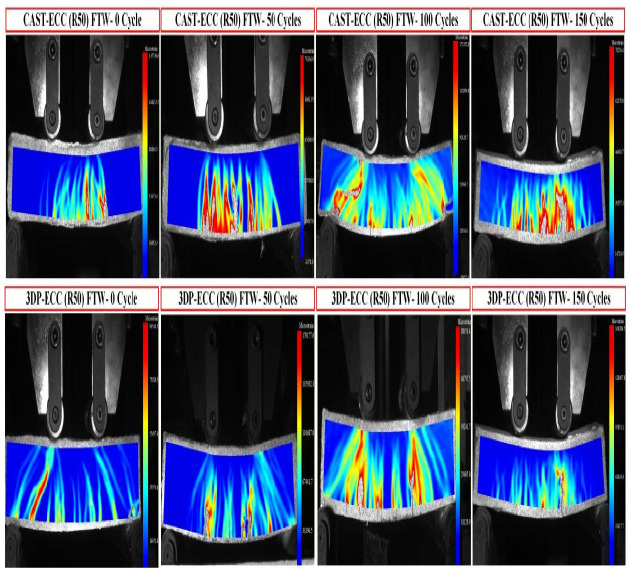
Full-field strain distribution obtained from digital image correlation.

Figure 14 presents the full-field maximum principal tensile strain distributions obtained from DIC analysis at the peak load state under different freeze–thaw cycles.

All strain contour maps were plotted using the same strain scale to enable direct comparison of strain localization and crack evolution. For uncycled Cast-ECC, the strain field was relatively uniform, and multiple fine cracks were distributed along the bending span. With increasing freeze–thaw cycles, tensile strain gradually concentrated near the mid-span region, leading to crack widening and stiffness reduction. Nevertheless, Cast-ECC still maintained relatively good structural integrity after 150 cycles because of its homogeneous matrix and effective fiber bridging.

By contrast, 3DP-ECC exhibited inherent anisotropic strain concentration along the interlayer regions even before freeze–thaw exposure. After repeated freeze–thaw cycling, this strain localization became more pronounced, and interlayer debonding gradually intensified. After 150 cycles, 3DP-ECC showed severe localized tensile strain and premature crack concentration, confirming that interlayer defects served as preferential paths for frost-induced crack propagation and flexural deterioration.

The residual flexural performance followed the order R50 > R25 > R0 > R75 > R100, confirming that the beneficial effect of YRS was content-dependent. For the R50 mixture, the peak load of Cast-ECC decreased from 9.89 kN to 6.5 kN after 150 freeze–thaw cycles, whereas that of 3DP-ECC decreased from 8.5 kN to 5.5 kN. The residual peak load of 3DP-ECC-R50 was therefore approximately 15.4% lower than that of Cast-ECC-R50. This difference demonstrates that printing-induced interlayer anisotropy reduced the ability of 3DP-ECC to maintain flexural resistance after freeze–thaw exposure.

Overall, Cast-ECC exhibited better flexural resistance and crack stability because of its more homogeneous microstructure, whereas 3DP-ECC was more vulnerable to interlayer debonding and anisotropic crack propagation. Moderate YRS replacement, especially 25–50%, improved freeze–thaw flexural durability by refining the pore structure and enhancing matrix compactness. However, excessive YRS incorporation aggravated structural heterogeneity and promoted crack localization. The statistical reliability of these flexural trends was supported by the ANOVA results in Section 3.7. The flexural strength models of Cast-ECC and 3DP-ECC showed *p*-values of 0.0012 and 0.0028, respectively, with R^2^ values of 0.9798 and 0.9689. The corresponding C.V. values were 1.26% and 1.17%, indicating good repeatability and limited experimental dispersion.

Since the detailed cooling and heating rates of the automatic freeze–thaw chamber were not independently recorded, the present study evaluates damage evolution mainly as a function of freeze–thaw cycle number rather than detailed thermal history. Future work should record the complete temperature–time profile within each cycle and quantify energy-related parameters, such as fracture energy, post-peak energy absorption, and toughness degradation, to further improve the reproducibility and mechanistic interpretation of freeze–thaw-induced flexural damage.

### 3.4. Microstructural Mechanism Revealed by XCT, MIP and SEM–EDS

To clarify the microstructural mechanisms underlying the freeze–thaw degradation and anisotropic mechanical response of Cast-ECC and Z-direction 3DP-ECC, XCT, MIP, and SEM–EDS analyses were conducted. XCT and MIP were used to examine the internal defect morphology and pore-structure parameters of representative R0, R50, and R100 mixtures. SEM observations were performed on the R50 mixture under different freeze–thaw cycles to investigate local crack evolution and interfacial morphology. SEM–EDS mapping was further used to evaluate local elemental redistribution before and after freeze–thaw exposure. These techniques provide complementary evidence at different scales; however, SEM–EDS results should be interpreted as local microstructural observations rather than as a fully quantitative chemical characterization of the entire matrix.

In the unfrozen state, the Cast-ECC matrix appeared relatively dense and homogeneous within the observed SEM field, and no obvious large microcracks were observed at the selected magnification. Nevertheless, the presence of nanoscale pores or defects outside the observed region cannot be excluded. For 3DP-ECC, the matrix also appeared generally compact, but the layer-by-layer printing process caused preferential alignment of hydration products and introduced interlayer interface defects [16,18]. With increasing freeze–thaw cycles, both Cast-ECC and 3DP-ECC exhibited progressive microstructural degradation [17,28].

After 50 freeze–thaw cycles, microcracks in Cast-ECC were mainly observed near particle boundaries, while the fine-particle filling and potential pozzolanic effect of YRS contributed to local matrix densification [20,21]. In contrast, damage in 3DP-ECC was more concentrated near the interlayer interfaces, accompanied by local microcracking and limited fiber pullout [18,19]. After 100 cycles, crack widening became more evident in both specimen types, and the loss of interlayer cohesion became more pronounced in 3DP-ECC [28]. After 150 cycles, localized degradation of hydration products and microcrack coalescence were observed in Cast-ECC, although PE fibers still bridged cracks and helped maintain matrix integrity [12,15]. For 3DP-ECC, freeze–thaw damage propagated preferentially along the interlayer regions, where hydration-product accumulation, microcracking, and interfacial debonding were more evident. Although PE fibers still contributed to crack bridging and stress redistribution, the weak interlayer regions remained the dominant paths for damage propagation [18,20].

XCT pore-threshold reconstruction and MIP results revealed clear differences in internal defect morphology between Cast-ECC and 3DP-ECC. In Cast-ECC, internal voids were relatively small, approximately spherical, and randomly distributed within the matrix. In contrast, 3DP-ECC exhibited more elongated and irregular voids, many of which were distributed along or near the interlayer regions. This difference can be attributed to the layer-by-layer extrusion process and the absence of vibration compaction during printing. The elongated voids and interlayer defects may serve as preferential paths for water ingress and local stress concentration, thereby accelerating freeze–thaw-induced microcracking and stiffness degradation.

The MIP results provided quantitative evidence for the pore-structure modification induced by YRS replacement. As shown in Table 9, the porosity of Cast-ECC decreased from 28.85% for R0 to 27.46% for R50 and 25.74% for R100, while the average pore size decreased from 25.41 nm to 24.54 nm and 22.41 nm, respectively. For 3DP-ECC, the porosity decreased from 29.24% for R0 to 27.65% for R50 and 27.67% for R100, while the average pore size decreased from 29.65 nm to 28.41 nm and 26.21 nm, respectively. These results indicate that YRS incorporation refined the pore structure and reduced the average pore size. However, the best mechanical and freeze–thaw performance was observed in the R50 mixture rather than in R100, suggesting that pore refinement alone does not fully determine durability. Excessive YRS replacement may increase matrix heterogeneity and weaken the balance among particle packing, interfacial bonding, and fiber-bridging efficiency.

Combining the XCT and MIP results, the improved freeze–thaw resistance of the R25–R50 mixtures can be attributed to the micro-filling effect and improved particle packing of YRS, which reduced visible internal defects and refined the pore structure. However, for 3DP-ECC, the layer-wise deposition process still introduced interlayer voids, anisotropic pore distribution, and preferential microcrack propagation. Therefore, the freeze–thaw degradation of 3DP-ECC should be interpreted as a coupled process involving matrix pore-structure refinement, interlayer defect evolution, anisotropic pore distribution, and microcrack propagation.

**Figure 15:**
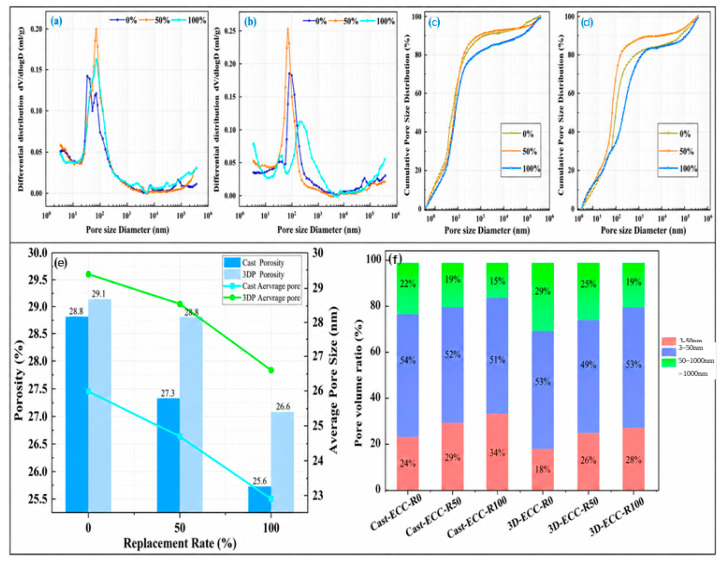
MIP pore-structure analysis of Cast-ECC and 3DP-ECC: (**a**) differential pore volume distribution of Cast-ECC; (**b**) differential pore volume distribution of 3DP-ECC; (**c**) cumulative pore-size distribution of Cast-ECC; (**d**) cumulative pore-size distribution of 3DP-ECC; (**e**) porosity and average pore-size comparison; and (**f**) pore-volume ratio distribution.

**Figure 16:**
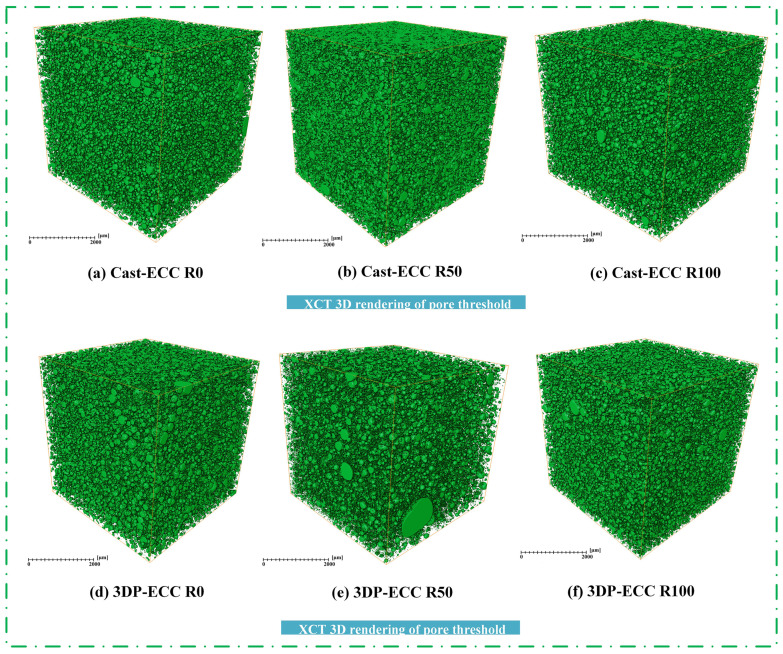
XCT analysis of voids and YRS threshold: (**a**) Cast-ECC R0; (**b**) Cast-ECC R50; (**c**) Cast-ECC R100; (**d**) 3DP-ECC R0; (**e**) 3DP-ECC R50; and (**f**) 3DP-ECC R100. Adapted from Ref. [29].

**Figure 17:**
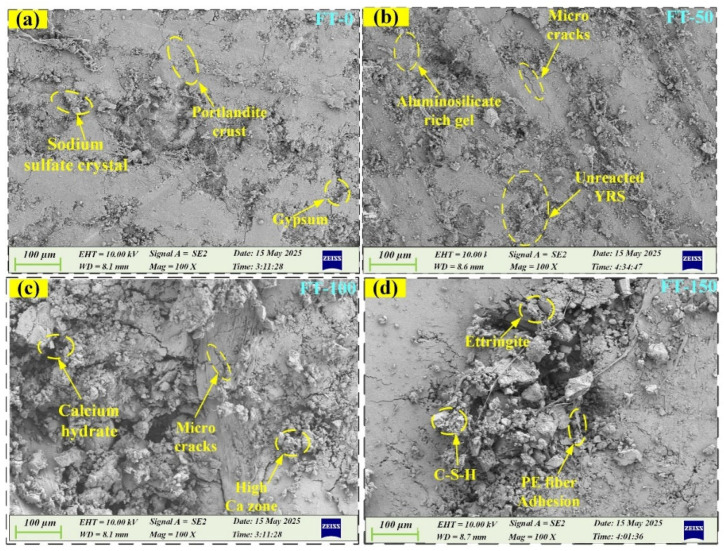
SEM images of Cast-ECC (R50) under freeze–thaw cycles: (**a**) 0, (**b**) 50, (**c**) 100, and (**d**) 150 cycles.

**Figure 18:**
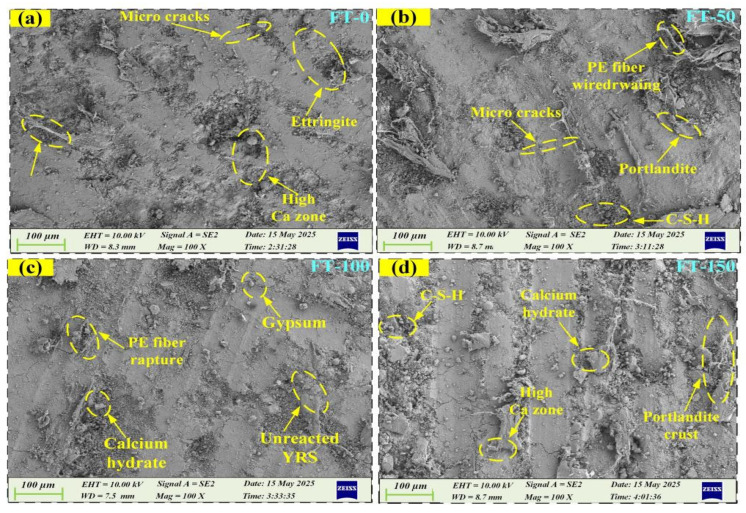
SEM images of 3DP-ECC (R50) under freeze–thaw cycles: (**a**) 0, (**b**) 50, (**c**) 100, and (**d**) 150 cycles.

**Figure 19:**
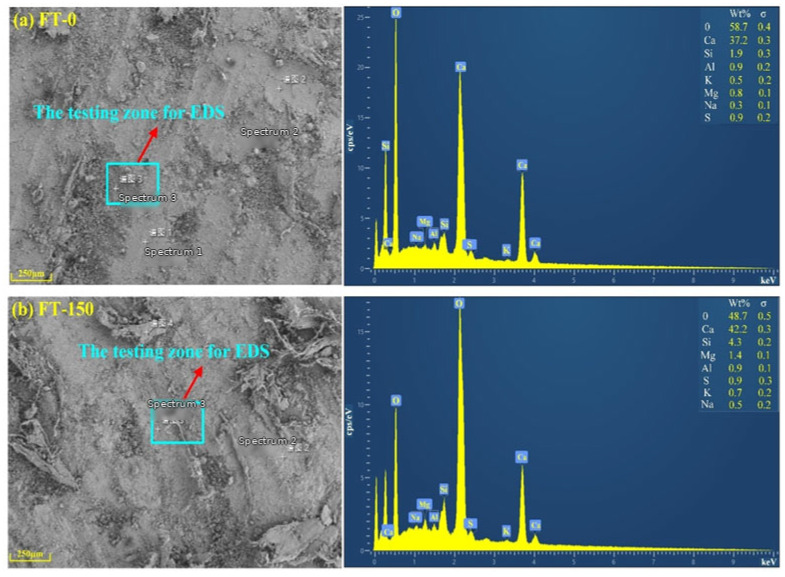
SEM–EDS analysis of Cast-ECC (R50) specimens: (**a**) FT-0 and (**b**) FT-150.

**Figure 20:**
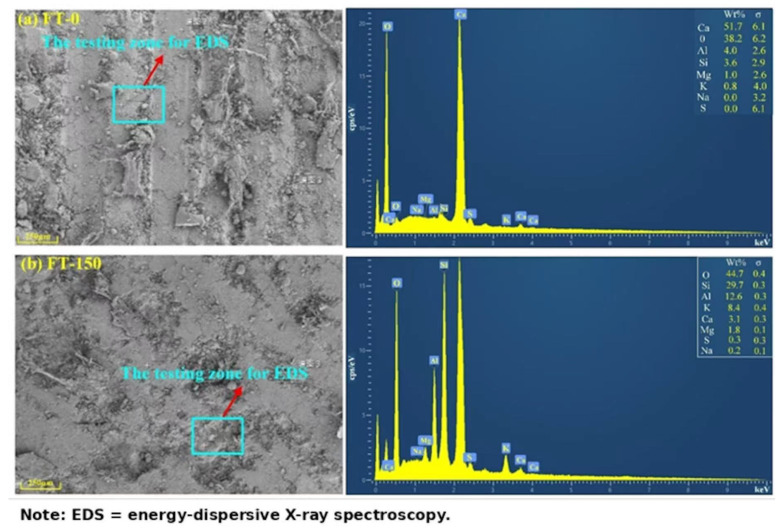
SEM–EDS analysis of 3DP-ECC (R50, Z direction): (**a**) FT-0 and (**b**) FT-150.

The MIP pore-structure analysis results of Cast-ECC and 3DP-ECC specimens are shown in Figure 15. The XCT reconstruction results of internal voids and YRS threshold segmentation are presented in Figure 16. Figure 17 shows the SEM images of Cast-ECC-R50 specimens under different freeze–thaw cycles, while Figure 18 presents the corresponding SEM images of 3DP-ECC-R50 specimens. The SEM–EDS analysis results of Cast-ECC-R50 and 3DP-ECC-R50 specimens before and after freeze–thaw exposure are shown in Figure 19 and Figure 20, respectively.

SEM–EDS observations from selected regions further suggest localized interlayer microcracking, calcium leaching, and elemental redistribution after freeze–thaw exposure, especially in 3DP-ECC. However, these observations should be regarded as local supporting evidence rather than as bulk quantitative phase analysis of the entire matrix. Overall, the combined XCT, MIP, and SEM–EDS results indicate that Cast-ECC maintained a relatively more homogeneous microstructure after freeze–thaw exposure, whereas 3DP-ECC was more vulnerable to interlayer damage and anisotropic crack propagation. This microstructural evidence supports the macroscopic degradation trends discussed in Section 3.2 and Section 3.3 and explains the difference in freeze–thaw performance between Cast-ECC and 3DP-ECC from the microscale perspective [17,19,28].

### 3.5. Discussion on the Mechanism of Freeze–Thaw Damage

The freeze–thaw degradation of ECC can be interpreted as a three-stage damage process governed by different dominant mechanisms. In the initial stage of 0–50 cycles, mass loss was limited and RDME degradation was relatively slow, indicating that damage was mainly associated with isolated microcrack initiation and early pore-water expansion. At this stage, internal defects had not yet formed continuous damage paths, and the matrix skeleton still maintained relatively good integrity.

In the development stage of 50–100 cycles, the decrease in RDME became more evident, suggesting that isolated microcracks and pores gradually connected and formed continuous damage paths [17]. The repeated expansion and contraction of pore water promoted crack propagation, while the deterioration of the pore structure accelerated stiffness loss. In the acceleration stage of 100–150 cycles, stiffness loss and mechanical strength degradation became more pronounced because crack coalescence, increased pore connectivity, and interlayer deterioration jointly reduced the effective load-bearing capacity of the material [17,28].

The dominant damage mode differed between Cast-ECC and 3DP-ECC. For Cast-ECC, freeze–thaw damage was mainly distributed within the matrix and around particle boundaries. This damage mode was primarily governed by matrix microcracking, pore-water expansion, and gradual deterioration of the fiber–matrix interface. For 3DP-ECC, however, the interlayer interfaces acted as preferential paths for water ingress, stress concentration, and crack propagation. Therefore, the damage evolution of 3DP-ECC should not be interpreted only as matrix deterioration, but rather as a coupled process involving matrix freeze–thaw damage, interlayer bond degradation, and anisotropic crack propagation.

In 3DP-ECC, the interlayer interface served as a structural weakness zone and played a dominant role throughout the damage process. This interface-dominated damage mode is fundamentally different from the degradation path of Cast-ECC, which is mainly controlled by distributed matrix microcracks [18,19,28]. The weak interlayer regions accelerated moisture migration and promoted localized damage accumulation under repeated freezing and thawing. Once interlayer defects were connected with matrix microcracks, the damage process shifted from dispersed microcracking to localized crack propagation, resulting in faster stiffness degradation and residual strength loss.

The incorporation of an appropriate amount of YRS mitigated freeze–thaw damage by optimizing particle gradation, improving matrix compactness, and refining the pore structure. Moderate YRS replacement reduced water migration channels and delayed the connection of internal defects, thereby inhibiting the development of freeze–thaw damage [18,20,27]. However, excessive YRS replacement may increase matrix heterogeneity and weaken cohesion, which promotes pore connectivity and accelerates crack propagation. Thus, the beneficial effect of YRS on freeze–thaw resistance is content-dependent rather than monotonic.

Overall, the freeze–thaw deterioration of YRS-ECC is controlled by the coupled evolution of pore-water expansion, matrix microcracking, fiber–matrix interfacial degradation, and, in the case of 3DP-ECC, interlayer bond deterioration. These microstructural and mechanical changes determine the random initiation and propagation of internal damage. To quantitatively describe this stochastic degradation process, a freeze–thaw damage evolution model is established and validated in the following section based on damage mechanics and the Weibull probability distribution.

### 3.6. Freeze–Thaw Damage Evolution Model and Validation

The aforementioned analysis of microstructural evolution and damage mechanisms indicates that the degradation of ECC in freeze–thaw environments is essentially a process of random initiation and propagation of internal microdefects. To quantitatively describe this stochastic damage behavior and enable long-term performance prediction, this section, based on damage mechanics theory, defines a damage variable characterized by the relative dynamic elastic modulus, and further introduces a two-parameter Weibull distribution [22,30] to establish a statistical model reflecting the freeze–thaw damage evolution laws of Cast-ECC and 3DP-ECC. The model is subsequently validated using data from four-point bending tests and dynamic elastic modulus measurements.

#### 3.6.1. Damage Variable

Within the framework of damage mechanics, the accumulation of internal microcracks, voids, and other defects can be regarded as a continuous damage field that evolves with the number of freeze–thaw cycles. To quantify this degradation process, a damage variable DD is defined, whose physical meaning is the loss ratio of effective load-bearing capacity induced by freeze–thaw action. Based on the relative dynamic elastic modulus results obtained in this study, D is expressed as follows:

(3) $$D=\frac{A_{∆}}{A_{i}}$$

(4) $$D=\frac{\bar{\sigma }-\sigma }{\bar{\sigma }}=1-\frac{\sigma }{\bar{\sigma }}$$

(5) $$D=\frac{\bar{E}-E}{\bar{E}}=1-\frac{E}{\bar{E}}$$

In these equations:

- D represents the degree of damage,
- $A_{∆}$ denotes the pore area or cracked zone after freeze–thaw deterioration,
- $A_{i}$ is the initial cross-sectional area,
- $\bar{\sigma }$ and σ are the true stress and effective stress, respectively,
- $\bar{E}$ is the initial dynamic elastic modulus,
- E is the residual modulus after n freeze–thaw cycles.

#### 3.6.2. Two-Parameter Weibull Damage Evolution Model

Cementitious composites are inherently heterogeneous; both Cast-ECC and 3DP-ECC specimens contain multi-scale defects such as capillary pores, interfacial voids, and microcracks. The random initiation and propagation of these defects impart significant statistical variability to the freeze–thaw degradation process. To accurately describe such stochastic behavior, this study adopts the two-parameter Weibull probability model, which has been widely used in the analysis of concrete fracture and durability, to characterize the damage evolution of YRS-ECC.

The probability of failure for the material is determined using Equation (6):

(6) $$P_{f}=\int_{0}^{n}f_{\Delta }\left(\delta \right)d\delta$$

Based on the two-parameter Weibull probability distribution model, f(N) is assumed to represent a probability density function following *N* freeze–thaw cycles.

(7) $$f\left(N\right)=\frac{\beta }{\eta }{\left(\frac{N}{\eta }\right)}^{\beta -1}exp\left[-{\left(\frac{N}{\eta }\right)}^{\beta }\right]$$

where η = scale parameter, representing the characteristic freeze–thaw life of the material, and β = shape parameter, defining the rate and uniformity of damage progression. By integrating this equation, the corresponding distribution function can be derived in Equation (8).

(8) $$F\left(N\right)=1-\exp\left[-{\left(\frac{N}{\eta }\right)}^{\beta }\right]$$

Following N freeze–thaw cycles, the probability of failure is given by

(9) $$P_{f}\left(N\right)=1-\exp\left[-{\left(\frac{N}{\eta }\right)}^{\beta }\right]$$

The probability of failure, $P_{f}\left(N\right)$, increases as the number of freeze–thaw cycles grows. At the initial state (*N = 0*), the material remains undamaged (D=0), while complete failure corresponds to D=1. The function reflects the gradual accumulation of microcracking, frost heaving, and internal stress concentration within the ECC matrix during cyclic freezing.

(10) $$D\left(N\right)=1-\exp\left[-{\left(\frac{N}{\eta }\right)}^{\beta }\right]$$

To facilitate regression analysis and parameter identification, the Equation can be linearized through a double logarithmic transformation.

(11) $$\mathrm{Ln}\left[\ln\frac{1}{1-D\left(N\right)}\right]=\beta \ln\frac{1}{\eta }+\beta \ln\left(\;N\right)$$

Let $\;Y=\ln\left[\ln\left(\;\frac{1}{1-D\left(N\right)}\right)\right]$, A $=\beta \ln\frac{1}{\eta }$, B=β, X=ln( N), then we get

(12) Y=A+BX

This equation satisfies D(0) = 0 at N = 0 and D → 1 as N → ∞, which is consistent with the boundary conditions of the damage variable, and can effectively describe the progressive stiffness loss resulting from the combined action of ice crystallization pressure and hydraulic pressure during the freeze–thaw process.

#### 3.6.3. Model Validation and Comparative Analysis

The damage variable was calculated from the measured RDME values and fitted using the Weibull-type statistical damage model. The fitted parameters and correlation coefficients are summarized in Table 10. The obtained R^2^ values ranged from 0.876 to 0.994, indicating that the model reasonably captured the overall trend of freeze–thaw damage evolution for both Cast-ECC and 3DP-ECC.

However, the fitting accuracy varied among different mixtures and printing directions. This variation suggests that freeze–thaw damage in 3DP-ECC cannot be interpreted solely as homogeneous matrix deterioration. Instead, it is also affected by localized interlayer defects, anisotropic pore distribution, and preferential crack propagation paths. Therefore, the Weibull model should be regarded as a statistical description of the overall RDME-based damage evolution rather than a complete physical representation of all local damage processes.

The fitted parameters listed in Table 10 include the Weibull shape parameter B (equivalent to β), the scale parameter η, and the coefficient of determination R^2^. The parameter B reflects the rate and uniformity of damage accumulation, while η represents the characteristic freeze–thaw life obtained from Weibull fitting. A higher B value indicates a more abrupt damage development stage, whereas a lower B value indicates more gradual and dispersed damage evolution.

The differences in B among Cast-ECC and 3DP-ECC specimens reflect differences in damage accumulation behavior. For Cast-ECC, the damage process was mainly associated with distributed matrix microcracking and pore-structure deterioration. For 3DP-ECC, the variation in B was more strongly influenced by interlayer voids, weak bonding regions, anisotropic pore distribution, and preferential crack propagation along the printed layers. These features increased the discreteness of damage development and caused the fitted parameters to vary among different printing directions and YRS replacement ratios.

(13) $$\mathrm{CAST}\text{-}\mathrm{ECC}\text{-}R0\;\;\;\;\;\;\;\;\;\;\;\;\;\;\;\;D\left(N\right)=1-\exp\left[-{\left(\frac{N}{289.89}\right)}^{1.18}\right]$$

(14) $$\mathrm{CAST}\text{-}\mathrm{ECC}\text{-}R50\;\;\;\;\;\;\;\;\;\;\;\;\;\;\;\;D\left(N\right)=1-\exp\left[-{\left(\frac{N}{334.31}\right)}^{2.09}\right]$$

(15) $$\mathrm{CAST}\text{-}\mathrm{ECC}\text{-}R100\;\;\;\;\;\;\;\;\;\;\;\;\;\;\;\;D\left(N\right)=1-\exp\left[-{\left(\frac{N}{1448.48}\right)}^{0.72}\right]$$

(16) $$3\mathrm{DP}\text{-}\mathrm{ECC}\text{-}Z\text{-}R0\;\;\;\;\;\;\;\;\;\;\;\;\;\;\;\;D\left(N\right)=1-\exp\left[-{\left(\frac{N}{455.34}\right)}^{1.41}\right]$$

(17) $$3\mathrm{DP}\text{-}\mathrm{ECC}\text{-}Z\text{-}R50\;\;\;\;\;\;\;\;\;\;\;\;\;\;\;\;D\left(N\right)=1-\exp\left[-{\left(\frac{N}{528.77}\right)}^{1.42}\right]$$

(18) $$3\mathrm{DP}\text{-}\mathrm{ECC}\text{-}Z\text{-}R100\;\;\;\;\;\;\;\;\;\;\;\;\;\;\;\;D\left(N\right)=1-\exp\left[-{\left(\frac{N}{241.10}\right)}^{3.27}\right]$$

**Figure 21:**
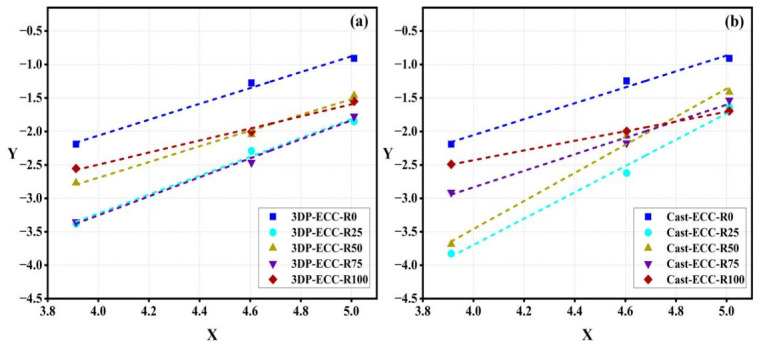
Weibull probability distribution fitting curves: (**a**) 3DP-ECC specimens with different replacement ratios; (**b**) cast-ECC specimens with different replacement ratios.

Comparison between the model-predicted damage evolution curves and the experimental data in Figure 21 shows good agreement. This confirms that the Weibull-based statistical damage model can effectively describe the progressive RDME degradation of ECC under freeze–thaw exposure. Nevertheless, the model parameters should be interpreted as comparative indicators of damage evolution rather than as absolute life-prediction parameters, because the fitting was based on a limited number of freeze–thaw intervals and did not explicitly incorporate detailed thermal history, pore connectivity, or interlayer defect geometry.

Overall, the Weibull-type damage model provides a useful analytical framework for comparing the freeze–thaw damage evolution of Cast-ECC and 3DP-ECC. It quantitatively captures the influence of YRS replacement and printing-induced anisotropy on stiffness degradation, while the physical interpretation of the fitted parameters should be combined with the XCT, MIP, SEM–EDS, and mechanical test results.

### 3.7. Statistical Analysis

A quadratic regression-based response analysis was conducted to evaluate the influence of YRS replacement ratio on the compressive and flexural strengths of Cast-ECC and 3DP-ECC. Since YRS was used to replace quartz sand on an equal-mass basis, the YRS content and quartz sand content were complementary variables rather than two fully independent factors. Therefore, the statistical interpretation was mainly based on the YRS replacement ratio instead of a conventional two-independent-factor mixture design.

The analysis of variance (ANOVA) results are summarized in Table 11. The results showed that all developed response surface models were statistically significant, with model F-values ranging from 31.18 to 110.62 and *p*-values lower than 0.05. For compressive strength, the Cast-ECC model showed an F-value of 110.62 and *p* = 0.0002, while the 3DP-ECC model showed an F-value of 45.33 and *p* = 0.0014. For flexural strength, the Cast-ECC and 3DP-ECC models showed F-values of 48.46 and 31.18, with *p*-values of 0.0012 and 0.0028, respectively. These results indicate that the YRS replacement ratio had a statistically significant influence on the mechanical properties of both Cast-ECC and 3DP-ECC.

The goodness-of-fit parameters for the strength models are presented in Table 12, further confirming the reliability of the regression models. The R^2^ values ranged from 0.9689 to 0.9910, while the adjusted R^2^ values ranged from 0.9378 to 0.9821, indicating that the models explained most of the experimental variability. The predicted R^2^ values ranged from 0.7818 to 0.9133, suggesting acceptable predictive capability. In addition, the model standard deviations were relatively low, ranging from 0.2392 to 0.3547, and the coefficients of variation were lower than 1.30% for all responses. Specifically, the C.V. values were 0.4181% for Cast-ECC compressive strength, 0.4687% for 3DP-ECC compressive strength, 1.26% for Cast-ECC flexural strength, and 1.17% for 3DP-ECC flexural strength. These low C.V. values indicate good repeatability and limited experimental dispersion.

The significant model terms also revealed the nonlinear influence of YRS replacement on mechanical performance. For Cast-ECC compressive strength, A, B, and A^2^ were significant terms. For 3DP-ECC compressive strength, A, A^2^, and B^2^ were significant. For Cast-ECC flexural strength, A, A^2^, and B^2^ were significant, whereas for 3DP-ECC flexural strength, A^2^ and B^2^ were the dominant significant terms. These results indicate that the effect of YRS replacement was not linear. Instead, moderate YRS replacement improved mechanical performance by optimizing particle packing, refining the pore structure, and enhancing matrix compactness, whereas excessive replacement weakened the beneficial balance between aggregate gradation and matrix cohesion.

It should be noted that the present statistical analysis was based on ANOVA and response surface modeling. Because the available dataset was organized primarily for regression model fitting, complete replicate-level raw data for all freeze–thaw conditions were not available for post hoc group-wise comparison. Therefore, Tukey’s HSD test and group-wise significance labeling were not included in the current analysis. Comparisons among individual mixture groups were consequently discussed mainly based on average trends, regression results, and C.V.-based repeatability rather than strict post hoc statistical grouping. Future work should include complete replicate-level datasets, error bars, and post hoc significance comparisons to further strengthen the statistical interpretation of differences among YRS replacement ratios.

## 4. Conclusions

This study investigated the freshwater freeze–thaw durability and anisotropic damage evolution of Cast-ECC and Z-direction 3DP-ECC incorporating different Yellow River sediment (YRS) replacement ratios. Based on mass loss, relative dynamic modulus of elasticity (RDME), residual mechanical properties, DIC observations, XCT, MIP, SEM–EDS analysis, Weibull-type damage modeling, and statistical analysis, the following conclusions can be drawn:

(1) Freeze–thaw deterioration of YRS-ECC exhibited a staged damage evolution process. During the early stage, mass loss remained limited, indicating that visible surface scaling was not the dominant damage mode. In contrast, RDME decreased earlier, suggesting that internal microcrack initiation and pore-water expansion preceded obvious surface deterioration. Therefore, RDME was a more sensitive indicator than mass loss for detecting early-stage freeze–thaw damage.

(2) Moderate YRS replacement improved freeze–thaw resistance by optimizing particle packing and refining the pore structure. The R25 and R50 mixtures showed slower RDME degradation and better residual mechanical performance than R0, R75, and R100. Among all mixtures, R50 exhibited the best overall residual compressive and flexural performance after 150 freeze–thaw cycles. This indicates that excessive YRS replacement may increase matrix heterogeneity and weaken mechanical durability, even though pore refinement can still occur.

(3) Compared with Cast-ECC, 3DP-ECC showed lower residual mechanical capacity, especially under flexural loading perpendicular to the printed layers. This behavior was mainly attributed to printing-induced interlayer defects and anisotropic pore distribution. Although RDME retention in some 3DP-ECC mixtures was comparable to that of Cast-ECC, the residual strength results and DIC strain fields confirmed that localized interlayer damage played a critical role in mechanical degradation.

(4) XCT and MIP results confirmed that YRS incorporation refined the pore structure and reduced visible internal defects. However, 3DP-ECC specimens still contained elongated voids and interlayer discontinuities associated with the layer-by-layer extrusion process. These features contributed to anisotropic freeze–thaw degradation. The optimal performance of R50 resulted from a balance among pore refinement, particle packing, interlayer compactness, and fiber-bridging efficiency.

(5) SEM–EDS observations provided local microstructural evidence of interlayer microcracking, hydration-product degradation, calcium leaching, and elemental redistribution after freeze–thaw exposure, especially in 3DP-ECC. These observations support the macroscopic degradation trends, but they should be interpreted cautiously as local evidence rather than as fully quantitative characterization of the entire matrix or pore system. Since XRD, FTIR, and TGA/DTG analyses were not conducted, hydration-product evolution and phase decomposition were not quantitatively determined in this study.

(6) The Weibull-type statistical damage model reasonably described the RDME-based freeze–thaw damage evolution, with fitted R^2^ values ranging from 0.876 to 0.994. The fitted parameters A and B were used as comparative indicators of damage evolution, where B corresponds to the Weibull shape parameter and reflects the rate and uniformity of damage accumulation.

(7) The ANOVA results confirmed the statistical reliability of the mechanical response models. The response surface models showed *p*-values lower than 0.05, R^2^ values ranging from 0.9689 to 0.9910, and C.V. values lower than 1.30%. These results indicate that YRS replacement had a statistically significant influence on the compressive and flexural performance of both Cast-ECC and 3DP-ECC, and that the experimental dispersion was limited.

(8) Overall, moderate YRS replacement, especially 25–50%, improved the freeze–thaw resistance of both Cast-ECC and 3DP-ECC by optimizing particle gradation, refining the pore structure, and enhancing matrix compactness. However, excessive YRS replacement increased matrix heterogeneity and accelerated degradation. For 3DP-ECC, freeze–thaw damage was governed not only by matrix deterioration, but also by interlayer defect evolution and anisotropic crack propagation.

Several limitations remain. First, XRD, FTIR, and TGA/DTG analyses were not conducted; therefore, hydration-product evolution and phase decomposition were not quantitatively characterized. Second, transport-related durability indicators, including water absorption, sorptivity, permeability, salt scaling resistance, chloride resistance, and salt freeze–thaw resistance, were beyond the scope of the present study. Third, complete replicate-level datasets for post hoc group-wise comparison were not available for all freeze–thaw conditions; therefore, Tukey’s HSD test and group-wise significance labeling were not included. Future work should incorporate complete error-bar analysis, post hoc statistical comparison, detailed thermal-history recording, and flexural energy parameters to further strengthen the quantitative interpretation of freeze–thaw damage evolution.

## Figures and Tables

**Table 1:** Basic properties of the cement.

Loss on Ignition (%)	Mixed Admixture (%)	Sulfur Trioxide (%)	Chloride ion (%)	Specific Surface Area (m^2^ · kg^−1^)	Stability	Coagulation Time (min)		28 d Strength (MPa)	
						Initial Setting	Final Setting	Flexural Strength	Compressive Strength
2.74	10.52	2.54	0.032	356	qualified	220	271	7.5	48.4

**Table 2:** Chemical composition of binders and fillers (%).

Type	$SiO_{2}$	Al_2_O_3_	Fe_2_O_3_	CaO	MgO	$SO_{3}$
Cement	24.85	8.96	4.03	51.39	3.72	2.42
Fly ash	54.41	24.52	5.86	5.37	0.87	-
Quartz sand	99.55	-	-	-	-	-
YRS	72.63	10.25	3.36	4.37	6.24	0.51

**Table 3:** Main technical indicators of YRS.

Apparent Density (kg·m^−3^)	Bulk Density (kg·m^−3^)	Saturated Surface Dry Water Absorption (%)	Specific Surface Area (m^2^ · g^−1^)
2646	1419	2.1	0.434

**Table 4:** Main technical indicators of PE fiber.

Diameter (µm)	Length (mm)	Tensile Strength (MPa)	Elastic Modulus (GPa)	Elongation at Break (%)	Density (g·cm^−3^)	Monofilament Cross-Section
22	12	3376	124	3	0.91	Approximately round

**Table 5:** Chemical and physical characteristics of attapulgite.

SiO_2_ (%)	Al_2_O_3_ (%)	Fe_2_O_3_ (%)	CaO (%)	Na_2_O (%)	K_2_O (%)	Whiteness (%)	pH	Moisture (%)	Specific Gravity	Specific Surface Area (cm^2^/g)
63	32	1.5	0.51	1.21	1.82	70	8	6.1	1.34	2112

**Table 6:** Mix design for 3D YRS ECC printing (kg/m3).

Replacement Rate (%)	Water	Cement	Quartz Sand	Fly Ash	YRS	Thickeners	Attapulgite	PE Fiber	Water Reducer
0	260	700	320	300	0	2.6	14	15.4	5.2
25	260	700	240	300	80	2.6	14	15.4	5.2
50	260	700	160	300	160	2.6	14	15.4	5.2
75	260	700	80	300	240	2.6	14	15.4	5.2
100	260	700	0	300	320	2.6	14	15.4	5.2

**Table 7:** Grouping of mechanical properties test.

Performance Index	Replacement Ratios	Specimen Size	Specimen Type	Number of Test Conditions	Replicates per Condition	Total Number of Specimens
**Compressive strength**	R0, R25, R50, R75 R100	50 mm × 50 mm × 50 mm	Cast-in-place Z Direction	40	3	120
**Three-point bending Strength**	R0, R25, R50, R75 R100	40 mm × 40 mm × 160 mm	Cast-in-place Z Direction	40	3	120
**Four-point bending Strength**	R0, R25, R50, R75 R100	40 mm × 40 mm × 160 mm	Cast-in-place Z Direction	40	3	120

**Table 8:** Freeze–thaw specimen mix designation.

Mix Designation	Specimen Type	Replacement Ratios	Number of Freezes–Thaw Cycles (N)	Freeze–Thaw Medium
**Cast specimens**				
**FT(0)**	Cast	R0, R25, R50, R75, R100	0	None
**FT(50)**	Cast	R0, R25, R50, R75, R100	50	Freshwater
**FT(100)**	Cast	R0, R25, R50, R75, R100	100	Freshwater
**FT(150)**	Cast	R0, R25, R50, R75, R100	150	Freshwater
**3DP-ECC** **(Z direction) specimens**				
**FT(0)**	3D-printed (Z direction)	R0, R25, R50, R75, R100	0	None
**FT(50)**	3D-printed (Z direction)	R0, R25, R50, R75, R100	50	Freshwater
**FT(100)**	3D-printed (Z direction)	R0, R25, R50, R75, R100	100	Freshwater
**FT(150)**	3D-printed (Z direction)	R0, R25, R50, R75, R100	150	Freshwater

Note: FT(0) denotes the absence of freeze–thaw cycles. FT represents freeze–thaw cycles conducted with freshwater. The letter ‘N’ signifies the count of freeze–thaw cycles.

**Table 9:** MIP pore-structure parameters of Cast-ECC and 3DP-ECC with different YRS replacement ratios.

Specimen	Porosity (%)	Average Pore Size (nm)	Pore Size Distribution Interval			Median Pore Size (nm)	Total Pore
			3 nm~50 nm	50 nm~1000 nm	>1000 nm		
Cast-R0	28.85	25.41	25%	51%	23%	7.85	23.65
Cast-R50	27.46	24.54	28%	53%	20%	6.74	22.41
Cast-R100	25.74	22.41	35%	49%	16%	5.65	22.27
3D-R0	29.24	29.65	19%	55%	28%	8.56	26.65
3D-R50	27.65	28.41	26%	51%	19%	8.23	27.95
3D-R100	27.67	26.21	29%	53%	20%	7.96	25.23

**Table 10:** Linearized Weibull fitting parameters and goodness of fit for freeze–thaw damage evolution.

Specimen	A	B	η	R^2^
CAST R0	−0.564	1.18	289.89	0.878
CAST R50	−0.745	2.09	334.31	0.876
CAST R100	−0.924	0.72	1448.48	0.926
3D-R0-Z	−0.926	1.41	455.34	0.921
3D-R50-Z	−1.418	1.42	528.10	0.994
3D-R100-Z	−1.375	3.27	241.10	0.9212

**Table 11:** ANOVA Summary for Mechanical Strength Models (Cast and 3D-Printed).

Response	Model F-Value	*p*-Value	Significant Terms	R^2^	Adj. R^2^	Pred. R^2^	Interpretation
**Compressive Strength—Cast**	110.62	0.0002	A, B, A^2^	0.9910	0.9821	0.9133	Excellent model fit; highly reliable
**Compressive Strength—3DP**	45.33	0.0014	A, A^2^, B^2^	0.9784	0.9568	0.7818	Strong model; good predictive accuracy
**Flexural Strength—Cast**	48.46	0.0012	A, A^2^, B^2^	0.9798	0.9596	0.9129	Excellent model reliability
**Flexural Strength—3DP**	31.18	0.0028	A^2^, B^2^	0.9689	0.9378	0.8265	Strong model; acceptable prediction

**Table 12:** Fit Statistics for Strength Models.

Response	Std. Dev.	Mean	C.V. (%)	Adeq Precision	Model Quality
**Compressive Strength—Cast**	0.2392	57.22	0.4181	33.70	Excellent signal; highly stable model
**Compressive Strength—3DP**	0.2616	55.81	0.4687	19.99	Strong signal; good predictive performance
**Flexural Strength—Cast**	0.3547	28.11	1.26	21.40	Excellent model stability
**Flexural Strength—3DP**	0.3081	26.44	1.17	16.55	Strong model and adequate prediction

## Data Availability

The original contributions presented in this study are included in the article. Further inquiries can be directed to the corresponding author.
